# Multipotent Mesenchymal Cells Homing and Differentiation on Poly(ε-caprolactone) Blended with 20% Tricalcium Phosphate and Polylactic Acid Incorporating 10% Hydroxyapatite 3D-Printed Scaffolds via a Commercial Fused Deposition Modeling 3D Device

**DOI:** 10.3390/biology12121474

**Published:** 2023-11-28

**Authors:** Nicola De Angelis, Andrea Amaroli, Alberto Lagazzo, Fabrizio Barberis, Pier Raffaele Zarro, Alessia Cappelli, Maria Giovanna Sabbieti, Dimitrios Agas

**Affiliations:** 1 Department of Surgical and Diagnostic Sciences (DISC), Unit of Implant and Prosthodontics, University of Genoa, 16132 Genoa, Italy; n.deangelis74@gmail.com; 2 Department of Dentistry, University Trisakti, Jakarta 10110, Indonesia; 3 Department of Earth, Environmental and Life Sciences (DISTAV), University of Genoa, 16132 Genoa, Italy; 4 Department of Civil, Chemical and Environmental Engineering (DICCA), University of Genoa, 16100 Genoa, Italy; alberto.lagazzo@unige.it (A.L.); fabrizio.barberis@unige.it (F.B.); 5 School of Biosciences and Veterinary Medicine, University of Camerino, 62032 Camerino, Italy; pierraffaele.zarro@studenti.unicam.it (P.R.Z.); alessia.cappelli@unicam.it (A.C.); dimitrios.agas@unicam.it (D.A.)

**Keywords:** mesenchymal stem cells, 3D printing, additive manufacturing, biopolymer, osteoinductive factors, personalized medicine

## Abstract

**Simple Summary:**

As highlighted by the ‘Global Burden of Disease Study 2019’ conducted by the World Health Organization, ensuring fair access to medical care through affordable and targeted treatments remains crucial for an ethical global healthcare system. Versatile polymers show promise, particularly in 3D printing, aiming to reduce costs and enhance healthcare accessibility, such as meeting dentistry’s demand for standardized osteoconductive products. It is essential to bridge biomaterial innovation with commercial printing technology. Our study emphasizes the metabolic behavior and lineage commitment of bone marrow-derived cells on two types of substrates: poly(ε-caprolactone) + 20% tricalcium phosphate (PCL + 20% β-TCP) and L-polylactic acid + 10% hydroxyapatite (PLLA + 10% HA). Despite the limitations of these polymers, these biomaterials effectively promoted osteoinductivity. Both substrates proved optimal for the commitment of bone marrow-derived multipotent mesenchymal cells (MSCs) to mature bone cells across different temporal sequences.

**Abstract:**

As highlighted by the ‘Global Burden of Disease Study 2019’ conducted by the World Health Organization, ensuring fair access to medical care through affordable and targeted treatments remains crucial for an ethical global healthcare system. Given the escalating demand for advanced and urgently needed solutions in regenerative bone procedures, the critical role of biopolymers emerges as a paramount necessity, offering a groundbreaking avenue to address pressing medical needs and revolutionize the landscape of bone regeneration therapies. Polymers emerge as excellent solutions due to their versatility, making them reliable materials for 3D printing. The development and widespread adoption of this technology would impact production costs and enhance access to related healthcare services. For instance, in dentistry, the use of commercial polymers blended with β-tricalcium phosphate (TCP) is driven by the need to print a standardized product with osteoconductive features. However, modernization is required to bridge the gap between biomaterial innovation and the ability to print them through commercial printing devices. Here we showed, for the first time, the metabolic behavior and the lineage commitment of bone marrow-derived multipotent mesenchymal cells (MSCs) on the 3D-printed substrates poly(e-caprolactone) combined with 20% tricalcium phosphate (PCL + 20% β-TCP) and L-polylactic acid (PLLA) combined with 10% hydroxyapatite (PLLA + 10% HA). Although there are limitations in printing additive-enriched polymers with a predictable and short half-life, the tested 3D-printed biomaterials were highly efficient in supporting osteoinductivity. Indeed, considering different temporal sequences, both 3D-printed biomaterials resulted as optimal scaffolds for MSCs’ commitment toward mature bone cells. Of interest, PLLA + 10% HA substrates hold the confirmation as the finest material for osteoinduction of MSCs.

## 1. Introduction

Over the past few years, significant advancements have been made in bone regeneration approaches, particularly in the field of dentistry, with a focus on creating alternative bone substitutes to autologous grafts. While allografts are commonly used, some patients may decline them for religious or ethical reasons [1]. Likewise, synthetic bone substitutes are extensively employed in dentistry, but they have drawbacks related to removal and exposure within the oral environment, which can increase the risk of infections and rejections [2,3]. During surgical procedures, shaping networks like titanium barriers can result in complexity and impact the outcome of the procedure [4]. Despite its invasiveness, autologous bone remains the preferred standard. Attention has been given to polymeric materials that are optimal for bone regeneration scaffolds. These biomaterials should possess characteristics such as low toxicity, high biocompatibility, biodegradability, good mechanical properties, and ease of shaping and disinfection. They should also have appropriate porosity and pore size to promote an ideal niche for the growth of new bone tissue and facilitate the formation of autogenous bone [5].

In this context, research is increasingly focused on identifying new technologies and materials to support medicine. However, often, these materials, although they settle on advanced protocols/ideas, still present low productivity and high manufacturing costs that limit market diffusion globally [6]. Therefore, alongside the development of increasingly innovative materials, the possibility of employing and modernizing familiar materials through new commercial technologies such as 3D printing could represent a parallel path of technological development aimed at meeting the need for ethical medicine.

Poly(ε-caprolactone) (PCL) is considered a promising candidate due to its elasticity and ease of fabrication, distinguishing it from various FDA-approved biodegradable polymers. However, using PCL for artificial constructs has two significant disadvantages. Its hydrophobicity makes protein matrix adhesion difficult, compromising cell adhesion and proliferation on the scaffold. Secondly, PCL lacks osteoinductive activity [7].

Polylactic acid (PLLA), or polylactide, is a synthetic absorbable biomaterial of polymer type, classified by the FDA as “generally recognized as safe” and approved for direct contact with biological fluids [8,9]. Specifically, PLLA is used as a bioabsorbable material at the skeletal site [10]. However, PLLA may be considered too fragile for applications requiring high plastic deformations at higher stress levels, such as those needed in dental applications [11].

Coupling agents have been employed to improve mechanical properties and surface adhesion between polymers and promote bone regeneration. In particular, polymer composites with beta-tricalcium phosphate (β-TCP) or hydroxyapatite (HA) have demonstrated improved mechanical properties and implementation of polymer bioactivity [12,13]. It should be noted that these materials have the potential to be employed in 3D printing or additive manufacturing, a promising technology that can revolutionize, accelerate, customize, and decentralize the production of medical devices made from innovative biomaterials [14]. 3D printing has the potential to modernize and simplify productive workflows and manufacturing techniques for temporary substitutes and prostheses, reducing energy consumption and carbon dioxide emissions compared to conventional processing technologies [15,16,17]. Additionally, the ability to customize treatments minimizes material waste, which often aligns with sustainable biomaterial practices [17]. While the utilization of bioresorbable polymers PCL and PDLA, supplemented with osteoconductive ceramics, is not a novel concept, their application in 3D printing has not been extensively explored. Therefore, it is fundamental to have identified the perfect balance of ceramic material quantity that enables printing using commercial 3D printers while eliciting an appropriate cellular biological response.

Overall, the development of 3D printing can reduce the impact of the healthcare sector on the environment. Essentially, the improvement of 3D printing technology can meet the needs of the global healthcare system in terms of ethical care from a socio-economic perspective, providing solutions for a sustainable, eco-friendly, circular economy and personalized medicine. Specifically, 3D printing technology can contribute to achieving the goals set by the World Health Organization (WHO) for the prevention and reduction of oral disease inequalities, which are key points in “The Global Burden of Disease Study 2019” report [18]. In a recent review, authors [19] have extensively explored the integration of 3D printing in biomedical fields. Their conclusion suggests that the primary applications encompass tissue engineering models, anatomical models, pharmacological designs, and models for validation. Comparable results were already identified a decade ago in other studies [20]. One of the prevalent application domains involves orthopedics and cranio-maxillofacial surgery, where customized anatomical components are crafted using various biopolymers [21,22]. Recent studies have highlighted the possibility of printing manufacturers with bioactivity targeted towards human multipotent bone marrow-derived mesenchymal cells (MSCs) and rat MSC cell lines, as well as MC3T3-E1 pre-osteoblasts [5,9,18]. However, despite promising results, such prints are often developed through laboratory prototypes or technology that is beyond the budget for widespread diffusion. The research aims to successfully marry surgical necessities, such as crafting precise and personalized implants, that eliminate the need for remodelling. These implants should also be biocompatible, osteoconductive, and inductive. Simultaneously, there is a need to minimize costs and facilitate broad integration into the everyday procedures of medical practitioners. This dual approach would help reduce expenses and make the treatment accessible across all social strata, even in technologically less advanced nations [23]. In a previous study [23], we demonstrated that a PCL compound supplemented up to 20% with β-TCP is suitable for commercial 3D printing using fused deposition modelling (FDM) and can withstand sterilization procedures according to ISO14937:200935 with a peracetic acid solution; it can also support osteoblasts’ homing and growth. In this work, we combined PLLA with up to 10% HA, and in line with what was previously done with the PCL + 20% β-TCP, compared to pure PCL, we showcased its printability through a commercial 3D printer. Subsequently, to assess potential post-production and post-sterilization modifications, we characterized the mechanical properties of PLLA combined with 10% HA. Lastly, we evaluated the PLLA + 10% HA and PCL + 20% β-TCP suitability of hosting and differentiating murine MSCs.

## 2. Materials and Methods

### 2.1. Sample Design and 3D Printing

The samples were prepared following the ISO 178 ASTM D790 standard. The dimensions of the test specimen for biological assays featured a thickness of 1 mm, while the diameter adhered to the standard dimensions of wells in multiwell plates, namely 11.5 mm in diameter. Consistent with our earlier research on PCL + 20% β-TCP [23], PLLA + 10% HA (synthetic hydroxyapatite) and PCL + 20% β-TCP were 3D-printed using a Prusa Mini LCD ^®^3D printer (Prusa Research a.s., Prague, Czech Republic), featuring a 0.2 mm nozzle size and printing temperatures of 175 °C or 110 °C, respectively. No additional post-printing procedures were conducted. Similar to the characterization process used for PCL + 20% β-TCP, the printed specimens underwent sterilization with a 2% peracetic acid solution following ISO14937:200935 standards.

#### 2.1.1. Statical Mechanical Tests (3 Points Bending Test Zwick Roell)

This test was carried out to assess the response of the PLLA + 10% HA samples to flexural stress. This mechanical analysis enabled the evaluation of critical factors, including the Modulus of Elasticity in bending, Flexural Strength, and the stress–strain response under flexural conditions. The assessments encompassed both non-sterilized composite polymers and those subjected to sterilization. Building upon the characterization methods previously published for PCL + 20% β-TCP, the testing machine employed was the Zwick Roell Z0.5 (ZwickRoell Group, Ulm-Einsingen, Germany).

The Zwick Roell equation governing the peak stress at the point of rupture is as follows:

$$\sigma_{max}=\frac{3FL}{2wh^{2}}$$

where:

*F*: Is the load at the bar center
*L*: Is the distance between the two lower supports
*w*: Is the width of the specimen
*h*: Is the thickness of the specimen

#### 2.1.2. Microscopic Morphological Analysis

Two distinct microscopic analyses were conducted using an optical microscope and a scanning electron microscope. In the optical microscope examination, test samples were scrutinized at 50× *g* and 100 × 0 magnifications using the Nikon Optical Microscope LV-100 (Nikon; Tokyo, Japan). The samples were simply positioned beneath the lens, without any supplementary treatment. For the scanning electron microscopy analysis, the samples were affixed onto metallic tape to facilitate the passage of the electron beam through the material. The apparatus employed for this analysis was the Hitachi S-2500 SEM (Hitachi, Tokyo, Japan).

### 2.2. MSC Collection and Cultures

Bone marrow-derived mesenchymal cells (MSCs) were obtained by flushing the femurs and tibiae of 3-month-old C57BL/6J male mice, as previously detailed [24]. These cells were subsequently seeded onto 3D-printed substrates (PLLA + 10% HA and PCL + 20% β-TCP) at a density of 15,000 cells/cm^2^. They were then cultured for varying durations (ranging from 7 to 21 days) in a minimum essential medium (α-MEM; Life Technologies, Monza, Italy) supplemented with 10% heat-inactivated fetal calf serum (HIFCS) (Life Technologies, Monza, Italy), penicillin, and streptomycin (Sigma Aldrich, Milano, Italy).

#### MSC Growth and Adhesion onto 3D Substrates

On the 7th day, cultures were utilized to assess cellular growth and adhesion to the substrates. Controls were established by cultivating the cells on culture dishes functioning as substrates.

An MTS (3-(4,5-dimethylthiazol-2-yl)-5-(3-carboxymethoxyphenyl)-2-(4-sulfophenyl)-2H-tetrazolium) assay was conducted to gauge cell viability. In brief, cells were incubated with CellTiter 96 Aqueous One Solution Reagent (Promega Italia, Milano, Italy) for 3 h, and the resultant-colored formazan was quantified by measuring absorbance at 490 nm using a TECAN reader (Tecan Italia s.r.l., Cernusco Sul Naviglio (MI), Italy).

In parallel, other cultures were washed with 0.1 M phosphate-buffered saline (PBS), pH 7.4, followed by fixation in 4% paraformaldehyde (PFA) [25]. Subsequently, these cultures were stained with 5% toluidine blue and captured using a light microscope (Zeiss Axioplan; Zeiss S.p.A., Milano, Italy). Image analysis was executed via NIH ImageJ software 1.54f [26]. Control samples consisted of cell-free substrates that underwent processing under the same conditions as the experimental samples.

On the 7th day, another group of cells underwent fixation and permeabilization with 0.3% Triton X-100 in PBS for 30 min. These cells were then incubated in 0.5% bovine serum albumin (BSA) diluted in PBS for 20 min, followed by exposure to a 4 × 10^−6^ mol/L phalloidin tetramethylrhodamine isothiocyanate (TRITC) conjugate (Sigma-Aldrich, Milan, Italy) diluted in PBS for 30 min at room temperature. Control samples consisted of cell-free substrates that underwent processing under the same conditions as the experimental samples.

For the investigation of TSG6 synthesis by MSCs, the fixed and permeabilized cells were incubated with a 1:60 dilution of rabbit anti-tumor necrosis factor-stimulated gene-6 (TSG6) (Abcam, Milan, Monza, Italy) in PBS. Subsequently, after washing, the cultures were subjected to a 1 h incubation at room temperature with a conjugated chicken anti-rabbit IgG Alexa Fluor 488 (Molecular Probes, Inc. Invitrogen, Monza, Italy) diluted 1:100 in PBS [27]. Following this, the cultures were washed and exposed to DAPI (diluted 1:1000 in PBS) for 45 min at 37 °C. The samples were then examined using a C2 Plus confocal laser scanning microscope (Nikon Instruments, Florence, Italy). The resulting microscope images were converted to a TIFF format and processed utilizing NIS-Elements imaging software (Nikon Instruments, Florence, Italy).

### 2.3. Cytokines and Chemokines Assay

The cytokine/chemokine profiles in supernatants of the MSC population cultured in 6-well culture plates and in the 3D-printed substrates were assessed by using Mouse Cytokine Array Panel A kit (R&D Systems, Milano, Italy) according to the manufacturer’s instructions.

#### MSC Differentiation

After 2 weeks of culture, MSCs were processed to evaluate the ability of the substrate to induce osteogenic differentiation. Cultured MSCs were in 6-well dishes with or without osteogenic (conditioned) medium (α-MEM containing 10% FCS, 25 μg/mL ascorbic acid-2-phosphate, 8 mM b-glycerophosphate, and 100 nM dexamethasone) and were used as controls. Cells, fixed and permeabilized, underwent overnight incubation at 4 °C with a rabbit anti-Pebp2aA/Runx2 antibody (1:50 dilution, Santa Cruz Biotechnology, Santa Cruz, CA, USA) or rabbit anti-Osterix antibody (1:50 dilution, Santa Cruz Biotechnology, Santa Cruz, CA, USA), both diluted in PBS.

After washing, the cultures were exposed to a conjugated chicken anti-rabbit IgG Alexa Fluor 488 (Molecular Probes, Inc. Invitrogen, Monza Italy) diluted 1:100 in PBS for 1 h at room temperature [27]. Then, the cultures were washed and subjected to a 45 min incubation at 37 °C with DAPI (diluted 1:1000 in PBS). Ultimately, the samples were scrutinized using a C2 Plus confocal laser scanning microscope (Nikon Instruments, Florence, Italy), with image processing executed as previously delineated.

Other MSCs, grown on the substrates for 21 days, were rinsed with PBS at pH 4.2. Control samples consisted of: (1) cell-free substrates; (2) MSCs cultured in 6-well dishes with or without osteogenic (conditioned) medium (α-MEM containing 10% FCS, 25 μg/mL ascorbic acid-2-phosphate, 8 mM b-glycerophosphate, and 100 nM dexamethasone). Control samples underwent processing under the same conditions as the experimental samples. They were subsequently immersed in a filtered 2% Alizarin red solution for 15 min at 37 °C. After washing, the substrates were observed via light microscopy (Zeiss Axioplan; Zeiss S.p.A., Milano, Italy). The quantitative assessment of Alizarin red staining was executed as previously specified [28].

### 2.4. Statistical Analysis

The statistical analyses were performed using MATLAB software (The Math-Works, Inc. MathWorks 1 Apple Hill Drive, Natick, MA, USA) and the means ± standard deviations were compared. Values of *p* < 0.05 were considered significant. Other data were analyzed by using one-way ANOVA followed by Tukey’s pairwise comparisons. Model *p*-value and sample number were labelled, with standard error depicted for each treatment site. Values of * *p* < 0.05 were considered significant. The results were representative of those acquired by independent experiments, which were repeated at least three times.

## 3. Results

### 3.1. 3D Printing

The samples of PLLA + 10% HA were successfully 3D-printed. The printing process demonstrated impeccable alignment with the .stl file, ensuring a consistent flow of composites through the nozzle. Various parameter settings facilitated a rapid and accurate cooling process, achieved without subjecting the print bed to additional thermal shock. To ensure the integrity of subsequent tests, no glue agents or adhesives were employed, thereby mitigating any potential contaminations that could have influenced the outcomes.

The samples of PCL + 20% β-TCP were also easily printed, confirming what was previously emphasized in our earlier research.

#### Statical Mechanical Tests—3 Points Bending Test Zwick Roell

This test was conducted on all the samples both before and after sterilization. This static mechanical test aimed to ascertain the composite polymer’s capacity to withstand the mechanical loads it might encounter during its implantation in the oral cavity.

The Zwick Z0.5 provided a stress–strain plot for each tested sample. This plot facilitated the extraction of critical parameters such as the Modulus of Elasticity in bending and Flexural Strength.

This methodology enhanced the statistical significance of the data by calculating the mean value and standard deviation of the aforementioned parameters. These values were then presented in a plot for immediate visual comparison.

Interestingly, the composite polymer PLLA + 10% HA exhibited varying mechanical properties before and after the sterilization process. Following sterilization, the PLLA + 10% HA samples exhibited a greater degree of deformation compared to the unsterilized counterparts, reaching a deformation of 10% (the maximum value predetermined during device setup).

**Figure 1:**
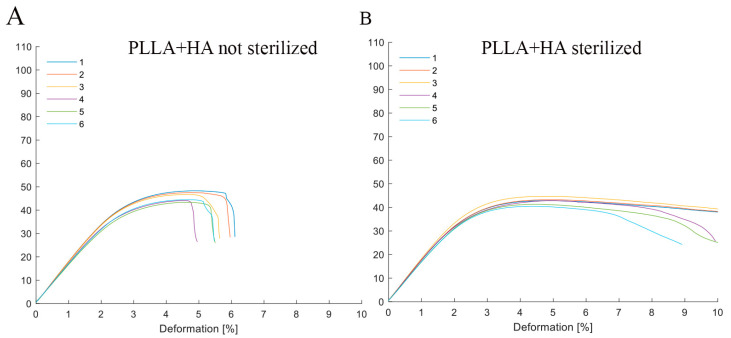
Representative diagrams for PLLA + 10% HA under non-sterilized conditions (**A**) and after sterilization (**B**), revealing a noticeable alteration, with the deformation percentage levelling off. The lines in the figure represent each tested specimen with different colors.

Table 1 and Table 2, along with Figure 1A,B, portray the mechanical parameters and the response of the material (PLLA + 10% HA) under the applied stress.

### 3.2. Microscopic Analysis

**Figure 2:**
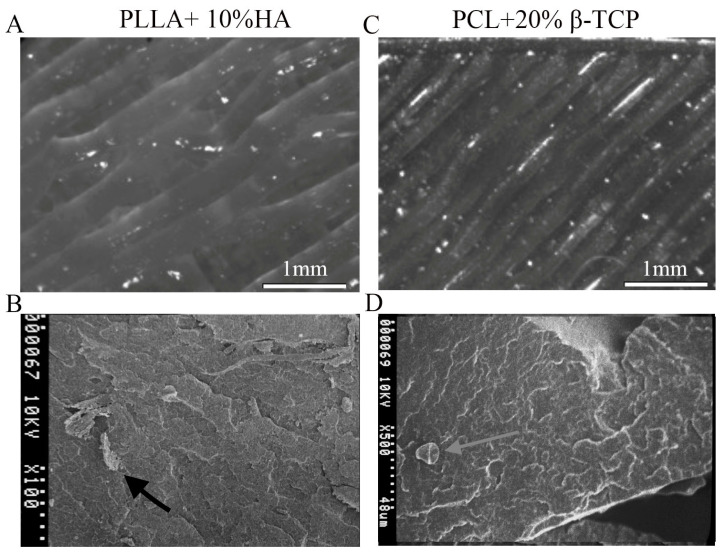
PLLA+ 10% HA after sterilization (50× *g* magnification). The even distribution of the printing lines is evident, with no surface anisotropy (**A**). HA granule within PLLA polymer prior to sterilization at 100× *g* magnification; no changes in granule dimensions occurred during the sterilization phase (**B**). (**C**) 50× *g*, (**D**) 500× *g*: β-TCP; no dimensional changes were noticed before and during the sterilization process. The arrows indicate the granules of hydroxyapatite and beta-tricalcium phosphate.

All samples underwent examination using the Nikon Optical Microscope. PLLA exhibited a consistent surface when viewed under the Optical microscope at a 50× *g* magnification. No irregularities, grooves, bubbles, or other imperfections were discernible, as depicted in Figure 2A. Furthermore, the sterilization process did not induce any changes to the surface. Subsequently, a more detailed analysis was carried out using SEM, limited to the composite polymers. This was undertaken to observe the dispersion of the ceramic material within the specimens and potential interactions post-sterilization. The outcomes of this evaluation showcased an even dispersion of the ceramic material and the absence of modifications or adjustments to the ceramic particles following sterilization. These results are illustrated in Figure 2B. Similar observations were made with PCL + 20% β-TCP (Figure 2C,D).

#### 3.2.1. MSC Adhesion and Expansion on the Substrates

**Figure 3:**
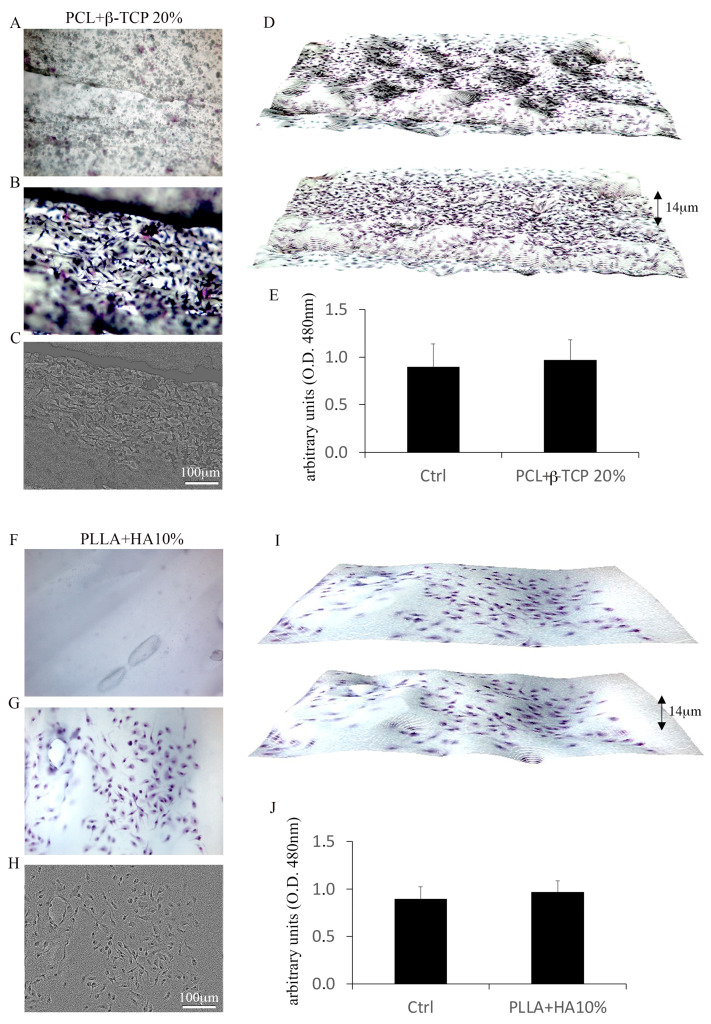
PCL + 20% β-TCP 3D-printed scaffolds in the absence of MSCs (**A**). Toluidine blue-stained MSCs on the meshes of PCL + 20% β-TCP scaffold (**B**) and surface plot (**C**); spatial illustration of the MSCs in different depths of the biomaterial (**D**); magnification 20× *g*. MTS assay highlights the efficacy of PCL + 20% β-TCP scaffold in hosting MSCs (**E**). Representation of PLLA + 10% HA 3D-printed scaffolds lack MSCs (**F**). Toluidine blue-stained MSC expansion on the PCL + 20% β-TCP scaffold (**G**) and surface plot (**H**); spatial distribution of the MSCs in different depths of the scaffold (**I**). Viability of MSCs cultured on PCL + 20% β-TCP 3D-printed scaffold (**J**).

**Figure 4:**
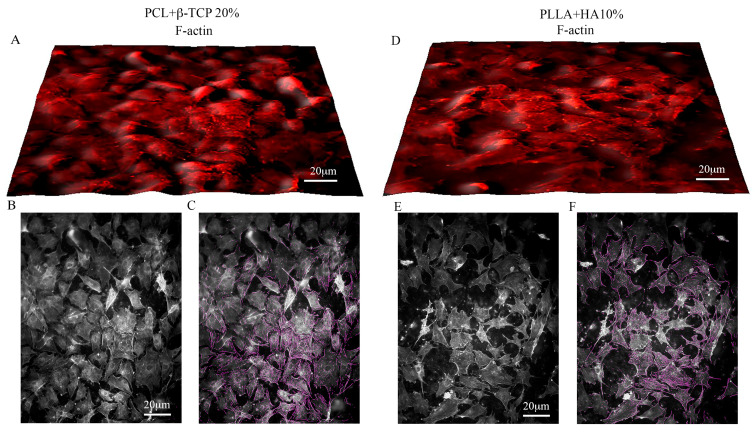
3D images of the phalloidin-labelled F-actin of MSCs over the PCL + β-TCP 20%-based substrate (**A**) or on the PLLA + 10% HA substrate (**D**). The surface plots of the substrates highlight the actin cytoskeleton (**B**,**E**). Note the MSC filopodia formation tagged with purple color (**C**,**F**). The open-source image processing software ImageJ [version ImageJ2 2.9.0/1.53t] was used for image analysis. Magnification 20× *g*.

**Figure 5:**
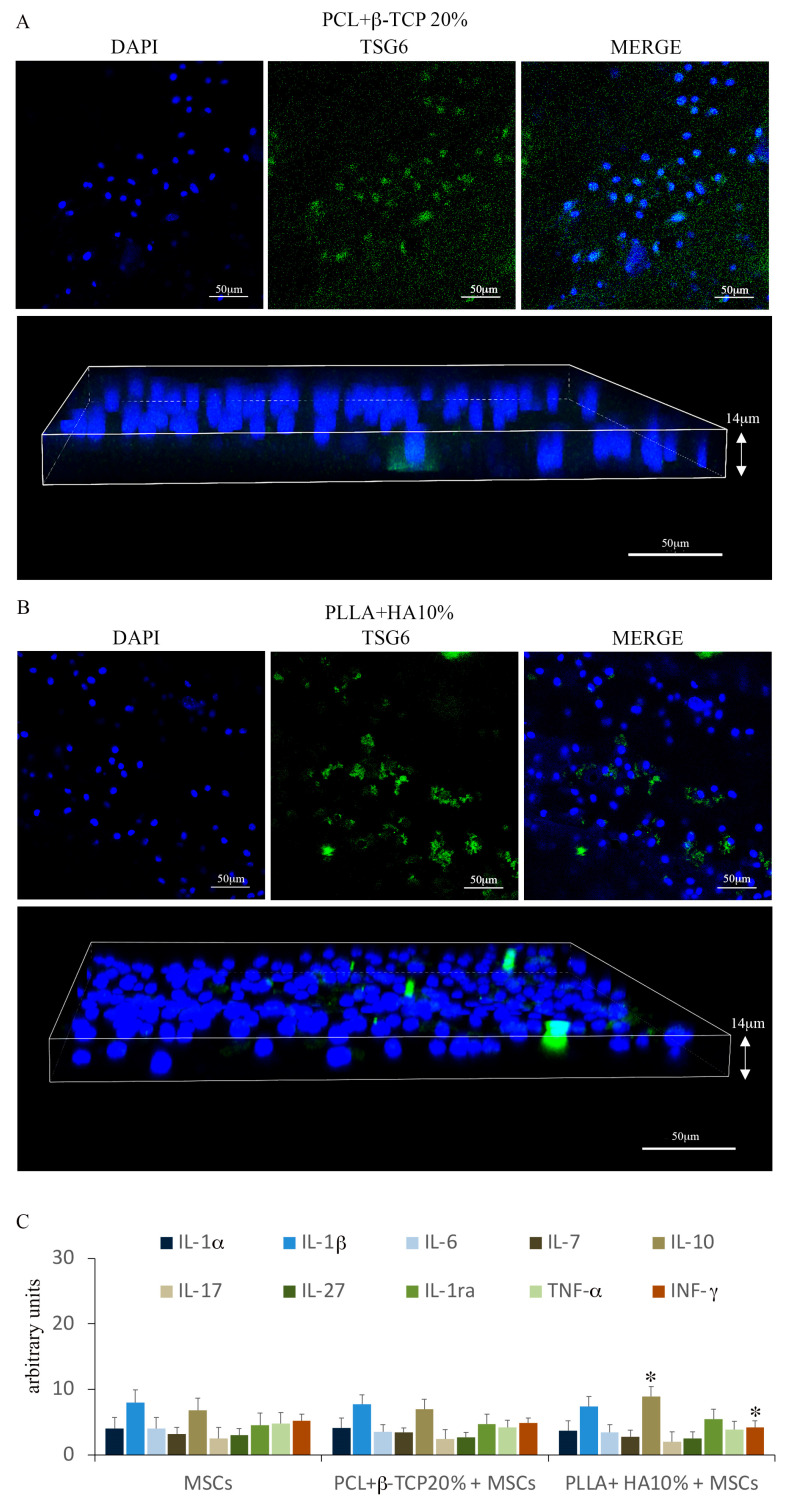
TSG6 expression on MSCs grown on the PCL + 20% β-TCP-based substrate (**A**) and on the PLLA + 10% HA-based substrate (**B**). Magnification 20× *g*. Cytokines and chemokines were analyzed from a medium gathered from MSCs cultured on a coverslip and on both biomaterials. The graphic represents the results of three independent experiments. Data were analyzed by using one-way ANOVA. Error bars represent ± SE (* *p* < 0.05 compared with MSCs) (**C**).

The adhesion and proliferation of MSCs on both substrates were assessed after 7 days of culture. The Toluidine blue assay vividly demonstrated the efficacy of PLLA + 10% HA- and PCL + 20% β-TCP-based biomaterials in hosting and supporting the multiplication of MSCs on their surfaces (Figure 3A–I). The distribution of MSCs within the meshes of both biomaterials appeared consistent, mirroring their viability measured via the MTS assay (Figure 3E,J). Evaluating the organization of the actin cytoskeleton serves as a benchmark for assessing cell behavior on substrates. The spatial arrangement of actin filaments orchestrates various homeostatic features, encompassing cell adhesion, proliferation, and differentiation. In this context, the spreading of MSCs’ actin filaments was scrutinized on both substrates using controlled low-strength material (CLSM) and a blend of 2D and 3D images. As depicted in Figure 4, PLLA + 10% HA- and PCL + 20% β-TCP-based biomaterials enhance the organization of actin filaments, which manifest as stress fibers running through the meshes of both substrates. Additionally, the comparison of multiple adhesion points and cell protrusions strongly supports the notion that these substrates significantly foster the establishment of cell–cell and cell–substrate junctions. It is widely recognized that MSCs play pivotal roles in immunoregulation, accomplished both through the release of paracrine factors acting on immune cells and through direct cell–cell interactions. Among the soluble factors released by MSCs, TSG6 holds notable immunomodulatory and anti-inflammatory functions. Intriguingly, notable labelling for TSG6 was observed in MSCs cultured on the substrates, while no labelling was observed in the cell-free substrates (Figure 5A,B). As a point of fact, secretome analysis of the experimental groups revealed that both 3D-printed substrates foster a low pro-inflammatory biofactor release. Notably, PLLA + 10% HA substrate denoted a slight but significant increase in IL-10 accompanied by a lower (but also significant) decrease in IFN-γ release compared with MSCs cultured in normal medium (Figure 5C).

#### 3.2.2. MSC Differentiation in Osteoblasts

**Figure 6:**
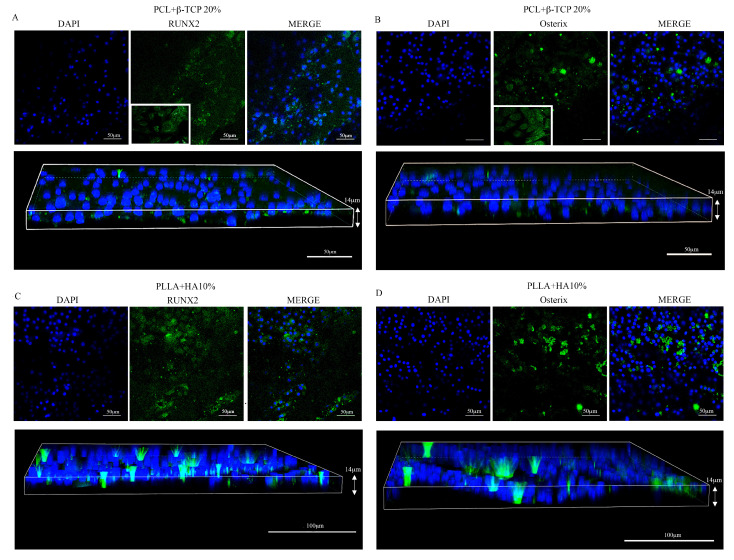
RUNX2 (**A**,**C**) and Osterix (**B**,**D**) expression on MSCs grown over the PCL + 20% β-TCP-based substrate and over the PLLA + 10% HA substrate, correspondingly. Note that both biomaterials stimulate the synthesis of the osteogenic transcription factors, albeit with slightly different levels. Magnification 20× *g*.

To determine if the 3D-printed biomaterials have the capacity to direct MSCs towards an osteogenic commitment, we examined the osteoinductive markers Runx2 and Osterix, which are expressed in the early and middle stages of pre-osteoblastic lineage. As depicted in Figure 6, both substrates effectively triggered the MSC synthesis of Runx2 and Osterix. Particularly, we observed a slightly higher but significant synthesis of both osteomarkers on PLLA + 10% HA compared with the PCL-based biomaterials. No labelling was detected on the substrates alone or in cells grown on the dishes in the absence of osteogenic medium. The Runx2 and Osterix expression in cells cultured in the presence of osteogenic medium was comparable to that found in cells grown on PCL-based biomaterials but lower with respect to that observed in cells maintained on PLLA + 10% HA substrates.

**Figure 7:**
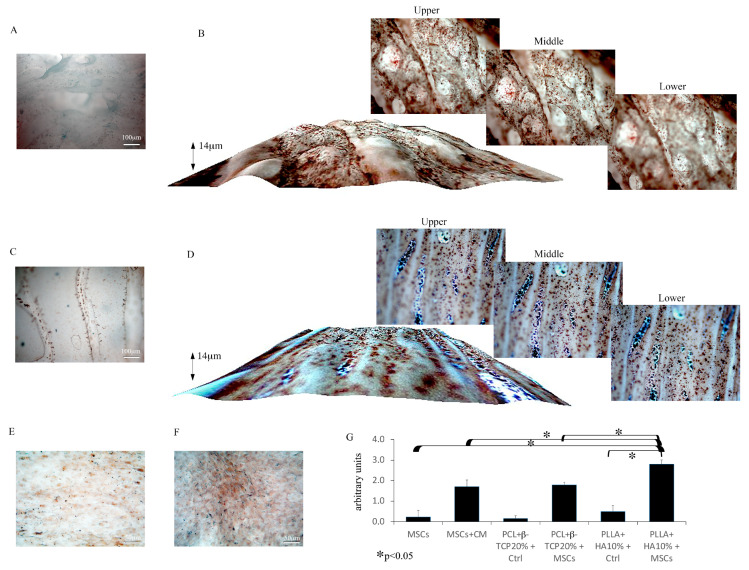
Alizarin red S-stained cell-free 3D-printed biomaterials (**A**,**C**). Alizarin red S staining on MSCs grown over the PCL + 20% β-TCP-based substrate (**B**) or the PLLA + 10% HA-based substrate (**D**). Note the important calcium deposition indicating the differentiation of MSCs toward osteoblasts displayed in image inserts (with different depths) or 3D spatial elaboration. Alizarin red S-stained MSCs cultured in α-MEM (**E**) or conditioned medium (**F**). Quantitative analysis of Alizarin red staining in all the experimental groups; note the slightly increased calcium deposition on the PLLA + 10% HA-based substrate hosting MSCs compared with the PCL + 20% β-TCP-based substrate hosting MSCs and the significantly increased level of PLLA + 10% HA-based group compared with the MSC-conditioned cultures (**G**); * *p* < 0.05. The open-source image processing software ImageJ [version ImageJ2 2.9.0/1.53t] was used for image analysis. Magnification 20× *g*.

Subsequently, Alizarin red S staining was conducted to gauge the deposition of calcium, a hallmark of bone cell lineage differentiation. The findings illustrated an augmentation in Alizarin red staining on both substrates harboring MSCs for 21 days (Figure 7B,D), with a maximal level found in MSCs grown on PLLA + 10% HA scaffolds (Figure 7D). While a slight Alizarin red S staining was also detected in the cell-free PLLA scaffold due to the presence of 10% HA (Figure 7C), this staining was definitely negligible compared to that observed in the 3D substrates hosting MSCs. On the other hand, the calcium deposition observed in cells plated with conditioned medium (partaking in control groups) (Figure 7F) was comparable to what was observed on PCL-based biomaterials (Figure 7D,G) but lower than what we detected on PLLA + 10% HA-hosting MSCs (Figure 7B,G). No significant Alizarin red S staining was observed in cells grown on culture plates without a conditioned medium (partaking in control groups) (Figure 7E).

**Figure 8:**
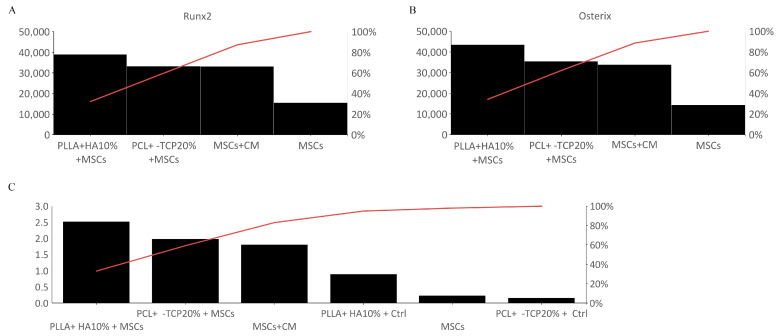
Quantization and percent variation of Runx2 (**A**) and Osterix (**B**) in all the experimental groups. Note the particularly evident percentage incrementation of both osteogenic markers on PLLA + 10% HA 3D-printed biomaterials. Fluorescence analysis from a pool of three different experiments was quantified by a Tecan Infinite fluorescence reader. The values were analyzed using Magellan v4.0 software and statistically analyzed. Quantitative analysis of Alizarin red staining in which the values are reported as percent variation of the experimental groups (**C**). Note the higher percent of calcium deposition on PLLA + 10% HA biomaterial compared with MSCs cultured with conditioned medium (CM) or even the PCL + 20% β-TCP-based substrate, which represents a considerable level of staining.

Overall, immunofluorescence quantitation of the tested biomaterials revealed that MSCs on PLLA + 10% HA display a greater expression of Runx2 and Osterix compared with MSCs cultured with or without conditioned medium and compared with MSCs cultured on PCL + 20% β-TCP. Our results depict the PLLA + 10% HA construct as ideal for MSC growth and differentiation toward an osteogenic bias. Additionally, the higher calcium deposition on PLLA + 10% HA biomaterial entirely buttresses the above posture (Figure 8).

## 4. Discussion

The concept of personalized or precision medicine has gained significant popularity over recent decades [29], and polymers stand as an ideal fusion of cost reduction, printing process simplicity, and potential for future widespread adoption. Moreover, the emerging research trend aims to extend into underdeveloped markets, making plastic materials an apt choice [30]. Nevertheless, it remains crucial to opt for materials that guarantee eco-friendly medical and dental treatments, as clinical services notably contribute to environmental pollution [31,32,33].

As a result, from a medical and dental standpoint, PLLA and PCL have been our preferred materials due to their individual or combined biodegradability [34,35]. The pattern of resorption is of paramount importance in a medical context, as it addresses the necessity to circumvent further surgeries for scaffold removal [36,37]. Simultaneously, from a biological perspective, the fabricated product should ensure an optimal lifespan to facilitate efficient osteoconduction and, if feasible, osteoinduction [38].

Delving into the specifics of our findings, the tests in compliance with ISO 178 ASTM D790 unveil that the PLLA + 10% HA composite displays distinct values in relation to tensile modulus and tensile strength when contrasted with the pure polymer behavior documented in the literature [39]. Notably, the recorded values for tensile modulus and tensile strength stood at 1.5–1.8 GPa and 43–48 MPa, respectively. The elongation at break was below 6%. These findings underscore the impact of even a modest proportion of HA in modifying the material’s mechanical characteristics. Conversely, in our prior analysis of PCL + 20% β-TCP [23], the composite exhibited statistically negligible alterations in behavior compared to pure PCL, registering values of 0.3–0.4 GPa and 13–15 MPa. It is worth noting that the PCL + 20% β-TCP composite underwent ISO 14937-200935 sterilization using a 2% peracetic acid solution without significant changes [23], in accordance with Italian national law n. 46/1997 and European Directive 93/42 concerning the decontamination and sterilization of critical devices.

Conversely, our data reveal that the PLLA + 10% HA composite exhibited differing behaviors pre- and post-sterilization with 2% peracetic acid. This is evidenced by an elongation at break that escalated with the extent of deformation subsequent to stress during flexural tests. This discernible alteration could potentially arise from liquid adsorption and might present a concern for an implant crafted from this material, given the heightened susceptibility to swelling.

Shifting our focus to biological characterization, our prior study showcased the capacity of MC3T3-E1 pre-osteoblasts to proliferate and thrive on PCL + 20% β-TCP while maintaining their morphological characteristics [23]. In this research, we demonstrate that both PCL + 20% β-TCP and PLLA + 10% HA substrates facilitate optimal adhesion, vitality, and expansion of mouse bone marrow-derived MSCs.

MSCs are extensively utilized for tissue repair, particularly in the context of bone regeneration, due to their unique attributes that extend beyond their inherent potential to differentiate into osteoblasts [40,41]. These adult stem cells, in fact, release a significant quantity of growth factors and cytokines that foster tissue repair. Furthermore, they release bioactive immunomodulatory molecules that mitigate inflammatory stimuli [42,43].

However, it is crucial to acknowledge that the behavior and activities of stem cells are fundamentally shaped by their microenvironment. Among the strategies aimed at enhancing the therapeutic release of stem cells, identifying a suitable extracellular matrix niche can be a possible pathway [44]. Our data underscore that both PCL + 20% β-TCP and PLLA + 10% HA stimulate the synthesis of anti-inflammatory cytokines/chemokines and the anti-inflammatory molecule TSG6, recently recognized for its protective, anti-inflammatory, and immunomodulatory properties [43,45]. Furthermore, our results align with prior studies indicating that 3D culture enhances the production of anti-inflammatory molecules, including TSG6 protein, compared to 2D culture [46].

As the proposed substrates were primarily developed to counteract bone loss, we assessed their osteoinductive potential by evaluating their ability to induce MSC differentiation into osteoblasts. The presented findings distinctly demonstrate the synthesis of Runx2 and Osterix, both critical osteoinductive markers [44,45], by MSCs cultivated on the scaffold for 14 days. This unequivocally signifies the efficacy of both PCL + 20% β-TCP and PLLA + 10% HA in promoting MSC commitment toward osteoblastic pathways. Furthermore, the substantial enhancement of Alizarin red S staining, a marker of mineralization [43], was clearly evident in both printed scaffolds. Notably, PLLA + 10% HA scaffolds exhibited an augmented capacity to effectively mimic an osteogenic microenvironment, adeptly guiding MSCs toward maturation into mature osteoblasts.

## 5. Conclusions

From a biological standpoint, both materials demonstrated favorable osteoconductivity and osteoinductivity. The latter was more pronounced in the PLLA + 10% HA composite. On the contrary, from a material perspective, although both composites can be easily printed using cost-effective Fused Deposition Modelling 3D Printers, the PLLA + 10% HA compound exhibits altered behaviors that hinder its disinfection with peracetic acid solution in accordance with ISO14937:200935. Both the polymeric matrices are highly biocompatible, and, in perspective, they may represent a viable option as scaffolds for bone regeneration. Due to its deformative behavior, PCL composite can be easily adapted to the bone defect in order to compensate for even minimal discrepancies between the project and the 3D-printed object; therefore, surgeons might prefer this latter composite over the PLLA compound. On the other hand, PLLA + 10% HA-printed biomaterial exhibited a significant capacity to commit MSCs toward osteoblastogenesis compared with the PCL-based tested compound and the MSCs cultured in a conditioned medium. In order to demonstrate the clinical efficacy of both materials, animal trials and/or human studies are needed.

## Figures and Tables

**Table 1:** PLLA + 10% HA before sterilization (E= elastic modulus; σ = compression; ε = elongation).

	E_f_	σ_fC_	σ_fM_	ε_fM_	σ_fB_	ε_ffB_
	MPa	MPa	MPa	%	MPa	%
PLLA P 1	1729.06	48.06	48.29	4.89	28.58	6.11
PLLA P 2	1667.91	47.39	47.51	4.75	28.42	5.96
PLLA P 3	1667.23	46.64	46.68	4.64	27.85	5.64
PLLA P 4	1623.66	44.07	44.09	4.50	44.09	4.50
PLLA P 5	1573.16	43.31	43.38	4.69	26.02	5.50
Mean PLLA	1642.91	45.65	45.74	4.69	30.27	5.53
SD_s PLLA	56.62	1.96	2.02	0.13	6.84	0.56

**Table 2:** PLLA + 10% HA after sterilization (E = elastic modulus; σ = compression; ε = elongation).

	E_f_	σ_fC_	σ_fM_	ε_fM_	σ_fB_	ε_ffB_
	MPa	MPa	MPa	%	MPa	%
PLLA P 1 S	1656.86	42.74	42.83	4.82	-	-
PLLA P 2 S	1667.39	43.11	43.25	4.87	-	-
PLLA P 3 S	1749.36	44.62	44.72	4.77	-	-
PLLA P 4 S	1702.16	42.54	42.80	4.92	25.66	9.93
PLLA P 5 S	1589.17	41.30	41.30	4.46	-	-
Mean PLLA S	1655.62	42.46	42.56	4.71	24.97	9.73
SD_s PLLA S	67.89	1.45	1.50	0.22	0.98	0.71

## Data Availability

The data that support the findings of this study are available from the corresponding author upon reasonable request.
